# Self-renewal of neural stem cells: implications for future therapies

**DOI:** 10.3389/fphys.2013.00049

**Published:** 2013-03-18

**Authors:** Susanna Raitano, Catherine M. Verfaillie, Anna Petryk

**Affiliations:** ^1^Stem Cell Institute, KU LeuvenLeuven, Belgium; ^2^Department of Pediatrics, University of MinnesotaMinneapolis, MN, USA; ^3^Department of Genetics, Cell Biology and Development, University of MinnesotaMinneapolis, MN, USA

In 2011, Remboutsika et al. (2011) published an elegant paper in which they demonstrated the unique importance of Sox2 in self-renewal of neural stem cells (NSC) *in vitro* and *in vivo*. They demonstrated that Sox2 helps maintain the cortical identity of NSC *ex vivo* as evidenced by the expression of Pax6 and the nestin-linked epitope RC2. Only the Sox2^+^ cells isolated from Sox^β−geo/+^ neurospheres were capable of generating secondary neurospheres while the Sox2^−^ fraction could not, confirming that only the Sox2^+^ cells can self-renew *in vitro*. Sox2^+^ neurospheres differentiated towards Tuj1^+^ cells with long axons like cortical neurons, while wild type and Sox^β−geo/+^ neurospheres derived-neurons developed short axons, showing that these cells had distinct developmental and differentiation potential.

When transplanted into mouse and chick embryos, wild type and Sox^β−geo/+^ cells generated neural crest cells while Sox2^+^ cells did not. Moreover, Sox2 overexpression in the neuroepithelium of chick embryo prevented neuroepithelial delamination and migration and restricted the contribution of neuroepithelium to the neural tube only, suggesting that Sox2 inhibits neural crest cell generation by blocking NSC differentiation.

## What are neural stem cells, and how can they be derived and maintained in culture?

NSCs (also named neural progenitor cells-NPCs) are multipotent stem cells generated during development when the neural plate folds to form the neural tube. NSCs give rise to all cells of the central nervous system. At the beginning of neurogenesis, neuroephitelial cells are replaced by radial glia, cells that can divide asymmetrically and differentiate into neurons, astrocytes and oligodendrocytes (Campbell and Gotz, 2002). Radial glia cells also act as a scaffold upon which neurons can migrate to specific locations in the developing brain (Rakic, 1972). In the adult brain, NPCs reside in the subventricular zone of the lateral ventricular zone and in the dentate gyrus of the hippocampus (Zhao et al., 2008).

NSCs/NPCs can be isolated from the cortex of mice and cultured *ex vivo* in non-adherent plates where they will aggregate and form neurospheres, composed of a mixture of stem cells, progenitors and differentiated cells. It is also possible to generate NPCs from mouse and human pluripotent stem cells (PSCs) (Uzzaman et al., 2005), using either adherent or suspension culture. Usually, factors that promote neural differentiation are added to the medium like retinoic acid (RA), Bone morphogenetic protein inhibitors (such as Noggin) and supplements such as N2 and B27.

The most common problem of *in vitro* neural differentiation is that it leads to a heterogeneous population of cells even when they are forced to a specific neural fate by specific growth factors. Therefore, various groups have developed methods to obtain pure population of NPCs. For instance, NPCs present in differentiating hPSCs that are CD184^+^CD271^−^CD44^−^CD24+ can be selected by fluorescence activated cell sorting (FACS) (Yuan et al., 2011). Alternatively, homogenous NPCs can be isolated based on the expression of polysialic acid-neural cell adhesion molecule (PSA-NCAM) (Kim et al., 2012). Yet another method is the use of molecular beacons, i.e., sequences that recognize specific regions of Sox2 mRNA, to FACS sort Sox2^+^ cells from mESCs as well as from neurospheres (Larsson et al., 2012). Remboutsika et al. (2011) described a novel approach using Sox2 lineage selection as a method to generate homogenous population of cortical NSCs.

## The role of Sox2 in neurogenesis

The Sox genes of the group B1 (Sox1, Sox2, and Sox3) are expressed widely in the central nervous system, and are implicated in neural development (Bergsland et al., 2011; Uchikawa et al., 2011). Sox2 is required for neural lineage commitment (Thomson et al., 2011; Wang et al., 2012) as it controls the proliferation and differentiation of fetal NPCs (Pevny et al., 1998; Wegner and Stolt, 2005). There is also evidence that Sox2 is expressed in differentiated cells of the adult brain (Kang and Hebert, 2012).

Genome-wide studies have shown that a significant number of Sox2 binding sites are unique to ESCs, and located in the vicinity of genes expressed in ESCs. In addition, a large number of binding sites are occupied by Sox2/3 in both ESCs and NPCs, located nearby neural genes and associated with bivalent histone domains. This is consistent with the notion that Sox2 in ESCs is a pioneer transcription factor that establishes transcriptional competence in ESCs for subsequent neural differentiation (Bergsland et al., 2011).

According to recent studies, adult somatic cells can be reprogrammed to mature cells or progenitor cells of non-related cell lineages. Ectopic expression of Sox2 leads to the generation of induced-neural like cells (iNCs) from human cord blood (CB) derived CD133^+^ cells, a process augmented by co-expression of c-Myc (Giorgetti et al., 2012). The CB-iNCs can fire action potentials and engraft the hypocampus *in vivo*. Likewise, fibroblasts and other somatic cells can be converted into induced neural stem cells (iNSCs) by transducing Sox2 alone or in combination with other transcription factors (Shi and Jiao, 2012).

The study by Remboutsika et al. was one of the first to demonstrate the importance of Sox2 in maintaining NSC undifferentiated, creating homogenous neurospheres, containing cells with the same spatiotemporal identity. This study and many others subsequently have detailed the role of Sox2 in establishing (through differentiation from ESC or de-differentiation from somatic cells) and maintaining cortical NSC features.

## The future of NSC research

Over the last 20 years, studies have been mostly focused on understanding which molecules and signaling pathways regulate differentiation, proliferation and migration of NSCs with an ultimate goal of applying cell replacement therapy for treatment of chronic neurologic diseases. The translational phase of this remarkable research has already begun. Several phase I/II clinical trials have been performed using purified NSCs for the treatment of amyothrophic lateral sclerosis, stroke, Batten disease, Pelizaeus-Merzbacher disease, high-grade gliomas (Trounson et al., 2011), spinal cord injury (Baker, 2011), cerebral palsy (Chen et al., 2013), and retinal diseases (Cramer and Maclaren, 2013). Even though it is still premature to say whether these therapeutic approaches will be effective, so far they appear to be safe. Further investigations are warranted to harness the full potential of NSCs.
